# Shaped by mountains, driven by climate: the rise of the Neotropical *Carex* sect. *Fecundae*

**DOI:** 10.1093/aob/mcag177

**Published:** 2026-07-07

**Authors:** Raúl Lois, Pablo García Moro, Ana Morales Alonso, Asunción Cano, Laurence J Dorr, Marcial Escudero, M Socorro González-Elizondo, Anton A Reznicek, Eduardo Ruiz-Sánchez, Jun Wen, Carmen Acedo, Pedro Jiménez Mejías

**Affiliations:** Área de Botánica, Departamento de Biodiversidad y Gestión Ambiental, Universidad de León, Campus de Vegazana s/n, León 24071, Spain; Área de Botánica, Departamento de Biología Molecular e Ingeniería Bioquímica, Universidad Pablo de Olavide, Carretera de Utrera km 1 sn, Sevilla 41013, Spain; Área de Botánica, Departamento de Biología Molecular e Ingeniería Bioquímica, Universidad Pablo de Olavide, Carretera de Utrera km 1 sn, Sevilla 41013, Spain; Área de Biodiversidad y Conservación, Departamento de Biología, Geología, Física y Química Inorgánica, Universidad Rey Juan Carlos, Calle Tulipán sn, Móstoles 28933, Spain; Departamento Académico de Botánica, Facultad de Ciencias Biológicas y Museo de Historia Natural, Universidad Nacional Mayor de San Marcos, Calle Germán Amézaga 375, Lima 1, 15081, Perú; Department of Botany (MRC-166), National Museum of Natural History, Smithsonian Institution, PO Box 37012, Washington, DC 20013-7012, USA; Department of Plant Biology and Ecology, Faculty of Biology, University of Seville, Seville 41012, Spain; CIIDIR Unidad Durango, Instituto Politécnico Nacional, Sigma 119, 20 de Noviembre II, Durango, Dgo. 34234, México; Department of Ecology and Evolutionary Biology, University of Michigan Herbarium, 3600 Varsity Drive, Ann Arbor, MI 48108, USA; Departamento de Botánica y Zoología, Centro Universitario de Ciencias Biológicas y Agropecuarias, Universidad de Guadalajara, Zapopan, Jalisco, 45200, Mexico; Department of Botany (MRC-166), National Museum of Natural History, Smithsonian Institution, PO Box 37012, Washington, DC 20013-7012, USA; Área de Botánica, Departamento de Biodiversidad y Gestión Ambiental, Universidad de León, Campus de Vegazana s/n, León 24071, Spain; Área de Botánica, Departamento de Biología Molecular e Ingeniería Bioquímica, Universidad Pablo de Olavide, Carretera de Utrera km 1 sn, Sevilla 41013, Spain

**Keywords:** Ancestor, Andes, biogeography, biome, Central America, Mexico, Neotropics, orogeny

## Abstract

**Background and Aims:**

*Carex* sect. *Fecundae* is a diverse group of sedges comprising 39 species, notable as a fully tropical lineage within a genus otherwise dominated by boreotemperate elements. Its distribution spans montane regions from Central Mexico to northern Chile and Argentina, where it occupies a variety of habitats. Despite its diversity and ecological importance, the section has remained understudied. The aim of this study was to generate the first nearly complete phylogeny of sect. *Fecundae*, to clarify evolutionary relationships, reconstruct its biogeographical history and identify the drivers underlying its diversification.

**Methods:**

We conducted phylogenetic analyses including 37 of the 39 known taxa of the section. Historical biogeographical reconstructions were used to infer dispersal pathways and colonization events. Climatic and ecological data were integrated to assess bioclimatic niche evolution, and morphological traits were evaluated for their potential role in diversification.

**Key Results:**

Analyses strongly support the monophyly of sect. *Fecundae*, with the exception of *Carex hebetata*. The phylogeny reveals evidence of rapid diversification associated with complex geological and climatic processes, particularly the uplift of mountain ranges in Mexico, Central America and the Andes. Biogeographical results suggest that the ancestor of the section dispersed long distance from North America to the Central Andes, followed by a progressive northward expansion into Central America and Mexico. Diversification coincided with Miocene global cooling and mountain uplifting, promoting adaptation to montane grasslands, shrublands and tropical moist broadleaf forests. Temperature and precipitation emerged as key variables shaping bioclimatic niches, with marked ecological differentiation between South American and Mexican lineages. Morphological features, such as stigma number and nutlet invaginations, appear to have influenced lineage divergence.

**Conclusions:**

The evolutionary history of sect. *Fecundae* reflects an interplay of long-distance dispersal, niche conservatism and local adaptation, enabling successful colonization of tropical montane environments. These findings contribute to a broader understanding of Neotropical montane biodiversity and highlight the dynamic processes shaping the evolution of tropical sedges.

## INTRODUCTION

Boreotemperate genera constitute a significant part of the Neotropical mountain flora (Antonelli and Sanmartín, 2011; Sklenář *et al.*, 2011). In general, boreotemperate plant lineages displayed a relatively conserved niche and found in the tropical mountains an environment analogous to their original habitat, which favoured their diversification (Hughes and Eastwood, 2006; Gehrke, 2018; Becker *et al.*, 2024). The migration of these boreotemperate elements primarily proceeded from north to south (e.g. Hughes and Eastwood, 2006; Cheng and Xie, 2013; Luebert and Weigend, 2014; Martín-Bravo *et al.*, 2019; Léveillé-Bourret *et al.*, 2020; Becker *et al.*, 2024), but movements in the opposite direction back to the north are also known (e.g. Sessa *et al.*, 2012). Although a stepping-stone migration mode mediating the closure of the isthmus of Panama has been a classical explanation for the entrance of the Boreal elements into the mountains of South America (von Hagen and Kadereit, 2001), there is growing evidence of the importance of long-distance dispersal (e.g. Vasile *et al.*, 2020). It has been confirmed that the timing of colonization is related to that of the elevation of the mountains, with lineages at higher elevations tending to be younger than those in lower areas of the Andes (Luebert and Weigend, 2014; McHenry and Barrington, 2014; Meseguer *et al.*, 2015).

The genus *Carex* L. (Cyperaceae) is one of the most diverse groups of flowering plants, comprising nearly 2000 species. To ease and compartmentalize the study of such high diversity, the genus has been divided into six subgenera and 62 sectional lineages, following phylogenetic evidence (Roalson *et al.*, 2021). *Carex* has a predominantly boreotemperate distribution, and it shows an inverse pattern of diversity: unlike the classic latitudinal gradient in species richness, in which diversity typically peaks in the tropics, *Carex* displays its highest species richness in the temperate regions of the Northern Hemisphere (Martín-Bravo *et al.*, 2019; Roalson *et al.*, 2021). This atypical biogeographical trend has been attributed to evolutionary adaptations that favoured temperate and cold environments, facilitating the expansion of the genus across high-latitude and montane regions, including cold-adapted niches in tropical mountain systems (Gehrke and Linder, 2009).

A key lineage illustrating the diversification of *Carex* in the Tropics is sect. *Fecundae*, the only section primarily restricted to tropical Americas and one of the few within the genus mostly confined to tropical environments (Roalson *et al.*, 2021; Lois *et al.*, 2025). Morphologically, the species of sect. *Fecundae* are readily distinguished by their mostly robust growth form, short-sheathing bracts, complex inflorescence architecture and the often dark-coloured glumes (Lois *et al.*, 2025). Its species are among the largest of the genus, including the longest flowering stem recorded for any *Carex* species (>3 m, in *Carex jovis* C.B.Clarke). Despite these distinctive traits, the section has historically received limited taxonomic attention, with only 9 of ∼20 recognized species included in earlier phylogenetic studies (see tree provided by Martín-Bravo *et al.*, 2019). Recent taxonomic revisions, however, have significantly expanded our understanding of the group, with the description of 21 new species, bringing the total to 39, based on extensive morphometric analyses (Lois *et al.*, 2023, 2025). Although extant molecular evidence points to the monophyly of sect. *Fecundae* (Roalson *et al.*, 2021), this conclusion was based on sparse sampling, underscoring the need for broader phylogenetic investigation. Furthermore, the precise position of the section within the broader *Carex* phylogeny remains unresolved.

Although numerous studies have examined *Carex* biogeography at deeper (subgeneric) phylogenetic levels (e.g. Martín-Bravo *et al.*, 2019; Benítez-Benítez *et al.* 2021*a*; García-Moro *et al.*, 2022), the shallow sectional-level biogeography remains relatively underexplored despite the central role of sections in the taxonomy of the genus (Lu *et al.*, 2021). This represents a notable gap, especially given that most documented north-to-south colonization events in *Carex* happened at the sectional level (Martín-Bravo *et al.*, 2019; Benítez-Benítez *et al.*, 2020; García-Moro *et al.*, 2022; Morales-Alonso *et al.*, 2025). Moreover, no comprehensive biogeographical study has yet focused on tropical *Carex*. To date, such studies have focused on groups of temperate distribution.

This study addresses that gap by examining the diversification of sect. *Fecundae* in the Neotropics, with particular emphasis on the role of geographical and macroecological requirements in shaping the evolutionary trajectory of this tropical lineage. By integrating phylogenomic, morphological, biogeographical and bioclimatic data, we aim to gain a better understanding of how boreotemperate lineages, such as *Carex*, diversify in tropical environments.

To elucidate the evolutionary history of sect. *Fecundae*, we: (1) built a time-calibrated genomic phylogeny; (2) reconstructed the biogeography and biome diversification history; (3) analysed the change of bioclimatic niches across the phylogeny; (4) inferred the evolution of relevant continuous and discrete taxonomic characters; and (5) performed diversification analyses and placed them in relationship to the number of stigmas and the invagination of the nutlets.

## MATERIALS AND METHODS

### Taxon sampling and DNA extraction

We included 89 samples from 71 *Carex* species, focusing on sect. *Fecundae* (55 samples from 37 species) plus 34 species as the outgroup. Plant material was obtained from both silica-dried field collections and herbarium specimens (Supplementary Data S1). This dataset includes nearly the entire known diversity of *Carex* sect. *Fecundae* (37 of 39 species, 95 % of the recognized species within the section), with the exception of *Carex melanosperma* Liebm. and *Carex spilocarpa* Steud, for which DNA extraction was unsuccessful. Almost all sequences were newly generated, except for some outgroup sequences that were obtained from Bioproject PRJNA553631 (Villaverde *et al.*, 2020).

Genomic DNA was extracted using a modified cetyltrimethylammonium bromide (CTAB) protocol (Doyle and Doyle, 1987) and quantified to ensure concentrations of ≥50 ng μL^−1^, adequate for library construction. Library preparation was performed by Arbor Biosciences (Ann Arbor, MI, USA) and the Plant Systematics Lab at Universidad Pablo de Olavide, using the NEBNext Ultra II DNA Library Prep Kit (New England Biolabs, MA, USA). DNA quality and concentration were assessed using a Qubit 2.0 fluorometer. Indexed libraries (500–700 ng per sample) were pooled in equimolar amounts in batches of ∼15 samples.

Target enrichment was carried out using the Cyperaceae-specific nuclear bait set developed by Villaverde *et al.* (2020), following Arbor Biosciences’ myBaits hybridization protocol. Sequencing was performed on an Illumina NovaSeq × 150 PE platform by Arbor Biosciences.

### Raw data processing

Raw sequencing reads were processed with Trimmomatic v.0.39 (Bolger *et al.*, 2014) to remove adapter sequences and low-quality bases using the following parameters: LEADING:3, TRAILING:3, SLIDINGWINDOW:4:15 and MINLEN:36. Cleaned reads were then analysed with HybPiper v.2.1.1 (Johnson *et al.*, 2016), which mapped reads to target loci using BWA v.0.7.17 (Li and Durbin, 2009). Contigs were assembled with SPAdes v.3.13.1 (Bankevich *et al.*, 2012), and intronic regions were removed by aligning contigs to reference exons.

Putative paralogues were identified and excluded using the built-in paralogue detection module in HybPiper. Multiple sequence alignments were generated with MAFFT v.7 (Katoh and Standley, 2013) using the default –auto setting. Sequence and alignment statistics were computed with HybPiper and AMAS (Borowiec, 2016), respectively.

### Phylogenomic analysis

We inferred phylogenetic relationships using both maximum likelihood and multispecies coalescent approaches based on the exon dataset.

Individual gene trees were reconstructed using IQ-TREE v.2.0 (Nguyen *et al.*, 2015; Minh *et al.*, 2020), with 1000 ultrafast bootstrap replicates (-b 1000). Spurious branches were pruned using TreeShrink (Mai and Mirarab, 2018) to reduce gene tree error. The resulting set of gene trees was used to estimate a species tree with ASTRAL (Zhang *et al.*, 2018), using the -t 2 option to calculate quartet support values. Support visualization was performed in R using the packages APE (Paradis and Schliep, 2019), ggtree, ggimage and treeio (Yu *et al.*, 2017; Wang *et al.*, 2020).

In parallel, all exon alignments were concatenated into a supermatrix using AMAS (Borowiec, 2016). The best-fitting partitioning scheme was selected with ModelFinder (Kalyaanamoorthy *et al.*, 2017) implemented in IQ-TREE, and a concatenated maximum likelihood phylogeny was inferred with 1000 ultrafast bootstrap replicates (-bb 1000). Resulting topologies were visualized and edited using FigTree v.1.4.4 (Rambaut, 2018).

To assess gene tree/species tree congruence, we calculated gene concordance factors (gCF) and site concordance factors (sCF) in IQ-TREE v.2.0, providing insights into the level of support across loci for each node in the species tree (Minh *et al.*, 2020).

### Divergence-time estimates

Divergence times were estimated using BEAST v1.10.4 (Suchard *et al.*, 2018). Given the size and complexity of the dataset, we adopted a ‘gene shopping’ strategy (Villaverde *et al.*, 2023) to reduce computational demands. Specifically, we selected 34 genes with quartet scores of >0.80, which were concatenated into a 78 372 bp matrix. Analyses were conducted under an uncorrelated relaxed lognormal clock model (Drummond *et al.*, 2006), using the GTR+Γ substitution model and a birth–death prior that accounts for incomplete taxon sampling (Stadler, 2009).

Three calibration points were applied: (1) the crown of *Carex* sect. *Phacocystis* Dumort (23.0–26.0 Mya, Early Miocene) (Martín-Bravo *et al.*, 2019); (2) the stem of sect. *Paniceae* (7.3–5.3 Mya, Late Miocene) (Martín-Bravo *et al.*, 2019); and (3) a secondary calibration on the *Limosae–Fecundae* split (95 % highest posterior density: 5.3–16.9 Mya) (Jiménez-Mejías *et al.*, 2016*a*; Martín-Bravo *et al.*, 2019).

Each BEAST run comprised 200 million Markov chain Monte Carlo generations, sampling every 10 000 generations. The first 20 million generations were discarded as burn-in, following Villaverde *et al.* (2023). Convergence and effective sampling were evaluated using Tracer v.1.7.1 (Rambaut *et al.*, 2018), ensuring effective sample size values of >200 for all parameters. Independent replicate runs were combined using LogCombiner v.1.10.4, and the maximum clade credibility tree was generated using TreeAnnotator v.1.10.

### Biogeographical history and biome diversification

We inferred ancestral geographical distributions using the BioGeoBEARS package (Matzke, 2018) implemented in RStudio. Both DEC and DIVALIKE models were tested, each with and without the founder-event speciation parameter (+J), given the ongoing debate surrounding its statistical validity (Ree and Sanmartín, 2018; Matzke, 2022). Although the fit is regarded as not comparable to that of models not accounting for the J paramater (Ree and Sanmartín, 2018), we still preferred also to test this model given the known propensity of *Carex* for long-distance dispersal and cladogenesis (Martín-Bravo *et al.*, 2019). Geographical ranges for taxa in *Carex* sect. *Fecundae* were coded based on 785 manually verified and georeferenced herbarium and field records across the Neotropics (Sanz-Arnal *et al.*, 2025), in addition to outgroup distributions in North America (NA). Biogeographical regions were delineated (modified from Morrone *et al.*, 2022), and species were assigned to them based on the occurrence records. We performed two different ancestral area reconstructions. The first used four broad areas to understand the dispersal and diversification to and within the Neotropics: (1) NA, North America (excluding Mexico) (only in the outgroup); (2) MEX, Mexico; (3) CA, Central America; and (4) S, South America. The second used eight finer-resolution areas to explore the migration routes within Tropical America: (1) NA, North America (excluding Mexico) (only in the outgroup); (2) SMO, Sierra Madre Oriental; (3) SS, Sierra Madre del Sur; (4) CTV, Trans-Mexican Volcanic Belt (Cinturón Transversal Volcánico Mexicano); (5) CCA, Cordillera Centroamericana (does not include Costa Rica and Panama mountain ranges); (6) TAL, Sierra de Talamanca (Costa Rica and Panama); (7) NAN, Northern Andes (south to Huancabamba Valley), including the marginal occurrences in the Guyana Massif; and (8) CAN, Central Andes (north to Huancabamba Valley, including the northernmost limit of the Southern Andes). An adjacency matrix was constructed to account for geographical connectivity among regions. Directly adjacent areas were assigned a value of one, whereas non-contiguous areas were given a value of 0.5. No regions were considered completely isolated (adjacency = 0), in line with the known capacity for long-distance dispersal in *Carex* (Martín-Bravo *et al.*, 2019).

The biome diversification study was based on terrestrial ecoregions of the world, based on the study by Olson *et al.* (2001) and modified by Benítez-Benítez *et al.* (2021*a*, *b*). We used the BioGeoBEARS package for the biome reconstruction analyses. To assign each species to its respective biome, we used the same georeferenced occurrence points. We considered four biomes: (1) Tropical and Subtropical Moist Broadleaf Forest; (2) Temperate and Tropical Coniferous Forests; (3) Montane Grasslands and Shrublands; and (4) Mediterranean Forests, Woodlands and Shrublands. As input data, we used the pruned phylogeny along with geographical presence/absence matrices for each biome. A marginal occurrence record of *Carex catamarcensis* C.B.Clarke ex Kük (Muñoz-Schüler *et al.*, 2023) within the Mediterranean biome in Chile was excluded from the analysis to avoid potential bias.

### Bioclimatic niche analysis

Occurrences for *Carex* sect. *Fecundae* were obtained from Sanz-Arnal *et al.* (2025). To characterize the bioclimatic niche of sect. *Fecundae*, we extracted bioclimatic variables (bio1–bio12) from WorldClim2 and an additional 16 variables from ENVIREM (Title and Bemmels, 2018). Variable selection followed the protocol of Benítez-Benítez *et al.* (2021*a*, *b*), which prioritizes variables contributing most to dataset variability while minimizing multicollinearity. The selected variables were mean diurnal range (bio2), precipitation warmest quarter (bio18), precipitation driest month (bio14), precipitation wettest quarter (bio16), temperature seasonality (bio4), aridity index and climatic moisture index. We conducted ancestral state reconstruction of ecological preferences using a dated singleton tree, pruning terminals to one tip per species, with the mean value per species for each selected variable. Analyses were implemented in the phytools v.0.7-7 package (Revell, 2012) in R. The adaptation of the climatic niche to selective optima was assessed using the *OUwie* function available in the R package *OUwie* (Beaulieu *et al.*, 2012; Beaulieu and O’Meara, 2016).

### Ancestral state reconstruction of discrete characters

We inferred ancestral states for nutlet invaginations and the number of stigmas. These are two conspicuous but poorly understood characters with different states in different species of sect. *Fecundae* (Lois *et al.*, 2025). To determine the best-fitting model of trait evolution, we used the fitDiscrete function from the Geiger v.2.0.7 package (Pennell *et al.*, 2014) in R.

Nutlet invaginations were coded as present (regardless of the number of invaginations) or absent. The number of stigmas was coded as either two or three, reflecting the observed variation within the section (Lois *et al.*, 2025). For both characters, we tested three evolutionary models: equal rates (ER), symmetrical rate (SYM) and all rates different (ARD). Model selection was based on the Akaike information criterion (AIC).

Ancestral state reconstructions were performed via Markov chain Monte Carlo sampling of the posterior distribution, generating 10 000 stochastic character maps using the make.simmap function in the phytools v.0.7-7 package (Revell, 2012).

### Evolution of continuous morphological traits

Plants belonging to sect. *Fecundae* are among the largest in plant height of the genus *Carex*. To approach the role of size in the evolutionary history of sect. *Fecundae*, we performed two sets of analyses over quantitative features. We examined seven continuous traits: culm length, blade length, blade width, number of spikes, first-order spike length, utricle length and nutlet length. For ancestral state reconstruction and comparative analyses, we had access to individual-level measurements (Lois *et al.*, 2025) and evaluated intraspecific variation for each trait. Because the ancestral state reconstruction methods implemented in the phytools package (Revell, 2012) require a unique value per phylogenetic tip (in this case, species), we confirmed that using mean trait values per species adequately represents species-level phenotypes, given the relatively low intraspecific variability in comparison to interspecific differences. To account for the evolutionary dynamics of these traits, we initially evaluated three alternative models of continuous trait evolution (Brownian motion (BM), Ornstein–Uhlenbeck (OU) and Pagel’s λ) using the *phylolm* package (Ho and Ané, 2014) in R. For each trait, we fitted intercept-only phylogenetic models and selected the best-fitting model based on the AIC (Supplementary Data Table S1).

First, we conducted ancestral state reconstruction of the traits using the dated singleton tree, with the mean value per species for each selected trait according to values provided by Lois *et al.* (2025) and as implemented in the phytools v.0.7-7 package (Revell, 2012) in R.

Second, we examined the correlation among different continuous traits across sect. *Fecundae* species. We conducted pairwise phylogenetic generalized least squares (PGLS) regressions. PGLS accounts for phylogenetic non-independence among species attributable to shared evolutionary history, allowing robust testing of trait correlations. We used the time-calibrated singleton ultrametric phylogeny derived from our dating analyses. Analyses were performed in R using the caper package (v.1.0.3; Orme *et al.*, 2013). A comparative.data object was created to link the phylogeny and trait data. For each trait pair, a separate PGLS regression was fitted via the pgls() function, with the phylogenetic signal parameter λ estimated by maximum likelihood. The value of λ ranges from zero (no phylogenetic signal) to one (Brownian motion). All continuous variables were log_10_-transformed prior to analysis to improve residual normality and homoscedasticity; residuals were visually inspected to confirm model assumptions. Statistical significance was assessed at *P* < 0.05. Results were interpreted as exploratory trait associations rather than tests of directional hypotheses.

Lastly, the strength of the phylogenetic signal in the most relevant continuous traits of sect. *Fecundae* (blade length, blade width, number of spikes, first-order spike length, utricle length, nutlet length, culm length and flower number) was quantified using both Blomberg’s *K* statistic and Pagel’s λ. Analyses were conducted in R using the phytools package. Prior to analysis, all trait data were log-transformed to improve normality. We then applied the phylosig function with the ‘K’ and ‘lambda’ methods to estimate the respective statistics. Corresponding *P*-values were obtained through simulation tests to assess the significance of the observed phylogenetic signal for each trait.

### Diversification analyses

Diversification rates along the phylogeny were estimated using a time-calibrated tree based on singleton representatives. We used BAMM (Bayesian analysis of macroevolutionary mixtures; Rabosky *et al.*, 2014) to explore rate heterogeneity in diversification across the phylogeny. The analysis was conducted in BAMM (Rabosky *et al.*, 2014) and processed with R using the BAMMtools package (Rabosky *et al.*, 2014), with priors set according to package recommendations via the setBAMMpriors function. A global sampling fraction of 0.95 was specified, reflecting the inclusion of 37 of the 39 known species. The reversible-jump Markov chain Monte Carlo algorithm was run with four independent chains, each for 10 000 000 generations, sampling every 10 000 generations. Additionally, posterior probability densities were calculated for the estimated anagenetic parameters.

We evaluated the fit of the two binary traits, stigma number (2 vs. 3) and nutlet morphology (invaginated vs. non-invaginated), using the binary state speciation and extinction model (BiSSE; Maddison *et al.*, 2007), comparing the full model with a constrained version with equal speciation rates. Additionally, we used the hidden state speciation and extinction model (HiSSE; Beaulieu and O’Meara, 2016) to account for the potential influence of unobserved (hidden) traits on diversification dynamics. Within the HiSSE framework, we tested three hypotheses: (1) the presence of a hidden state associated with stigma number; (2) the presence of a hidden state associated with nutlet morphology; and (3) the presence of separate hidden states for both traits. All analyses were conducted in R using the diversitree package.

### Evaluation of nutlet invagination and area of occupancy

The invagination in the nutlet remains a poorly understood feature of enigmatic functionality despite being a character reported from different *Carex* groups. Because it is a character present in the *Carex* diaspore, it might, presumably, point to a role in the dispersal and/or establishment of populations. We explored the potential relationship between species distribution area and the presence/absence of nutlet invaginations. Considering that each category (not invaginated or invaginated utricles) includes a reasonable balanced number of species (15 and 13, respectively), we used a PGLS (Orme *et al.*, 2013) model, with area of occupancy as the response variable and nutlet invagination as a binary predictor, to determine whether species with larger distribution areas are more likely to exhibit invaginated nutlets. To account for shared evolutionary history among species, analyses incorporated a pruned phylogenetic tree corresponding to the species with complete data. Phylogenetic logistic regressions were performed using the phylolm R package, applying two estimation methods: IG10 (importance sampling) and MPLE (maximum penalized likelihood estimation). These methods model the association between nutlet invagination and distribution area while accounting for phylogenetic relationships. Estimated coefficients, standard errors and Wald test *P*-values were examined. The parameter α estimated in the models represents the strength of phylogenetic signal in the residuals.

Areas of occupancy were calculated (in square metres) using the convex hull method based on the occurrences from Sanz-Arnal *et al.* (2025). Narrow endemic species represented by only a single occurrence point were excluded from the analysis. In addition, *Carex chordalis* Liebm. was identified as an outlier because its area was >1.5 times the interquartile range above the third quartile (Q3). Accordingly, we removed it from the analysis to avoid skewing the results.

## RESULTS

### Phylogenomic analyses

Newly produced sequences are available in the Bioproject PRJNA1346283. Out of the initial 525 genes studied, we excluded 41 because they were identified as potential paralogues or were not recovered for most species, resulting in a final supermatrix of 985 620 bp for *Carex* sect. *Fecundae*. The average number of reads per sample was 7 210 277, ranging from 202 302 to 26 704 098, with an average mapping success of 60.39 % (range: 7.29–91.50 %). From the recovered genes, 188 919 sites (19.20 %) were variable, and 72 063 (7.31 %) were parsimony informative. ModelFinder identified GTR+F+I+G4 as the best-fitting evolutionary model based on both the AIC and the Bayesian information criterion.

*Carex* sect. *Fecundae* was recovered as monophyletic in our phylogeny (Fig. 1) with strong bootstrap support (BS = 100). It was nested in a grade of species that are usually placed within sect. *Phacocystis* Dumort: *Carex barbarae* Dewey, *Carex crinite* Lam. and *Carex nebrascensis* Dewey. The section diverged early into three distinct clades: (1) a two-stigmas clade comprising only South American Andean endemics; (2) a morphologically heterogeneous clade containing *Carex lehmanniana* Boott, *Carex via-aquae* Jim.Mejías *et al.* (two-stigmas) and *Carex galeottiana* C.A.Mey. (three-stigmas); and (3) a three-stigmas clade of mostly Central American and Mexican species. Most internal nodes throughout sect. *Fecundae* topology showed high support (BS > 90). However, substantial gene-tree discordance was detected across certain branches of the phylogeny. Gene concordance factors (gCF) ranged from 0.58 to 75.05, and site concordance factors (sCF) ranged from 25.56 to 90.31. In the sect. *Fecundae* clade, 45 % of branches presented >10 gFC, and 89 % presented >33 sCF. Discordance was more prevalent along backbone branches than near the tips or among outgroup taxa (Fig. 1). In particular, despite full bootstrap support (100 %) for all backbone nodes within sect. *Fecundae* (Fig. 1), four clades exhibited notably low concordance values (<10 % gCF and <33 % sCF): (1) the clade incuding *Carex breedlovei*, *C. tojquianensis*, *C. lapazensis*, *C. caribea*, *C. mikasae* and *C. mesoamericana* (gCF = 3.33, sCF = 30.2); (2) the clade including *C. breedlovei*, *C. tojquianensis*, *C. lapazensis*, *C. caribea* and *C. mikasae* (gCF = 1.55, sCF = 25.6); (3) the clade including *C. chordalis*, *C. jovis* and *C. purepecha* (gCF = 4.66, sCF = 30.2); and (4) the clade including *C. tuberculata*, *C. transmexicana* and *C. catrina* (gCF = 0.77, sCF = 32.9).

**Fig. 1.**
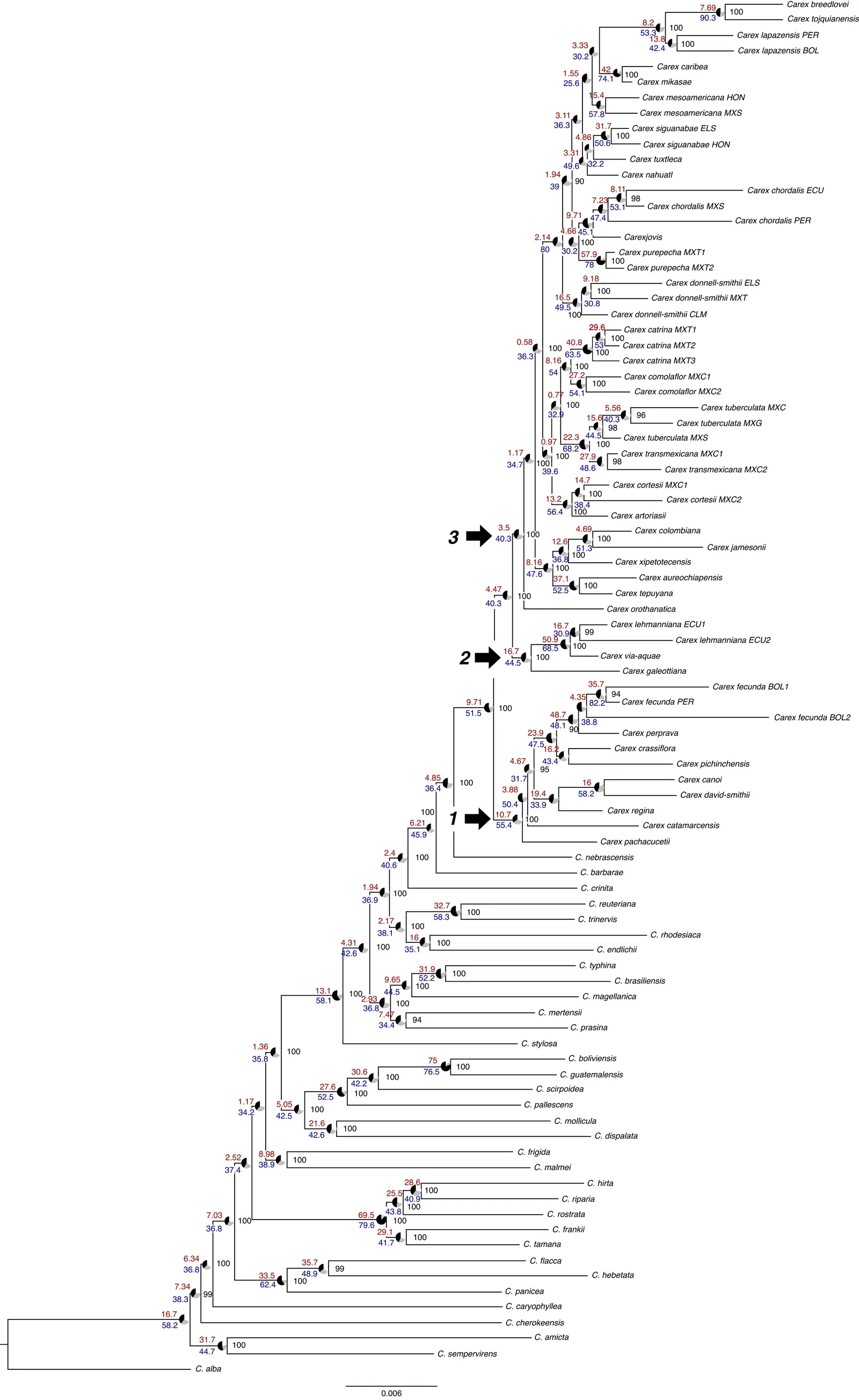
Maximum likelihood phylogenetic reconstruction using IQ-Tree2 of 37 species from *Carex* sect. *Fecundae* and including 89 samples (484 nuclear genes exon supermatrix). Branch support values of >90 % are shown in black to the right of each one. gFC and sFC are shown above (red) and below (blue) of each branch, respectively. Pie charts showing quartet support values at the nodes (black, loci agreeing with the main topology; grey, loci supporting first alternative topology; white, loci supporting alternative topologies).

Notably, *C. hebetata*, previously assigned to sect. *Fecundae*, was recovered outside the section in a well-supported clade (BS = 99) with *Carex flacca* Schreb. Additionally, *Carex nebrascensis* (sect. *Phacocystis* Dumort) was recovered as the sister lineage to the sect. *Fecundae*, with full bootstrap support (BS = 100).

### Divergence-time estimates

The phylogeny inferred using BEAST was largely congruent with the topology recovered by IQ-TREE. The stem age of *Carex* sect. *Fecundae* was estimated at ∼10.28 Mya, with a 95 % highest posterior density interval of 7.06–13.71 Mya, situating its origin in the Late Miocene. The three main clades also diverged before the end of the Miocene, and the cladogenetic events articulating the backbone of each clade happened around the Mio-Pliocene boundary. Most shallow nodes are placed within the Pliocene, with only three pairs of species, each encompassing at least one narrow endemic species, seemingly dated to the Pleistocene (Fig. 2).

**Fig. 2.**
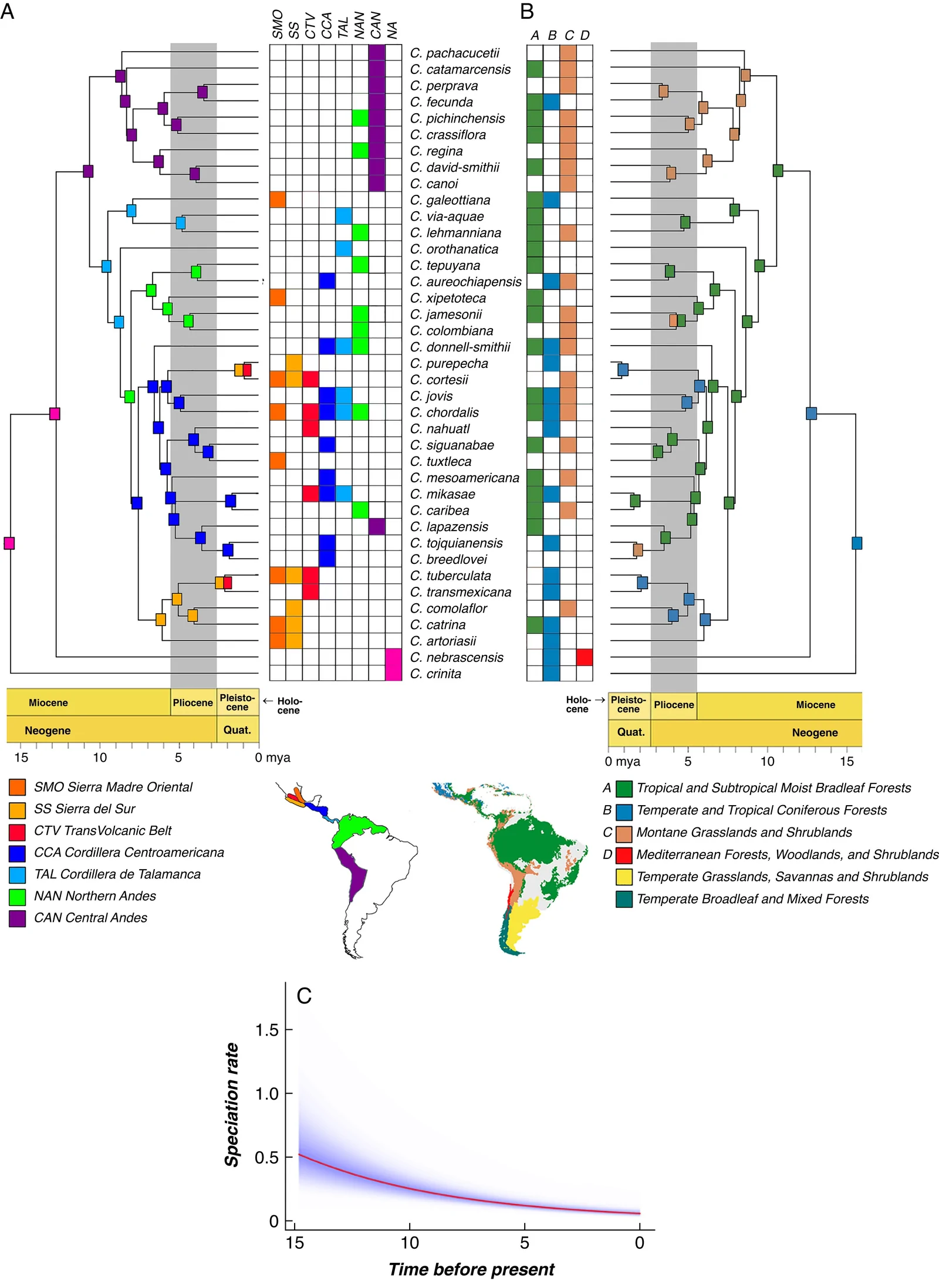
(A) Biogeographical reconstruction and divergence-time analysis of *Carex* sect. *Fecundae* following our eight-area DEC+J results. Colours correspond to the key in the map. (B) Reconstruction of biomes (following Benítez-Benítez *et al.*, 2021*a*, *b*) based on the constrained reduced species tree calibration of sect. *Fecundae* under the DEC+J model as implemented in BioGeoBEARS. The tree displays the most probable biome or combination of biomes on each node with coloured squares. Colours correspond to the key in the map, regardless of whether the biome has been coded or not. (C) Net diversification rate through time. The red line displays the mean net diversification rate, and the shaded range indicates its reversible-jump Markov chain Monte Carlo confidence interval.

### Biogeographical history and biome diversification

The DEC+J model provided the best fit for our data in both the eight-area reconstruction (log-likelihood = −126.68, AIC = 259.36; Fig. 2A) and the four-area reconstruction (log-likelihood = −78.78, AIC = 163.56; Supplementary Data S2). Both analyses consistently inferred South America, in particular the Central Andes, as the ancestral area of *Carex* sect. *Fecundae* (eight-areas analysis). This implies a long-distance dispersal event from North America in relationship to the origin of the section. From now on, we will focus on the more detailed eight-area reconstruction. The two-stigmas clade was inferred to have originated in South America. In contrast, the remainder of the section was traced back to an ancestral area in the Sierra de Talamanca in southern Central America, where at least two cladogenetic events took place. From this region, the three-stigmas clade expanded northwards, with the most recent common ancestor of the group inferred to have originated in the Cordillera Centroamericana. This group subsequently diversified extensively and expanded into the Mexican mountain ranges, namely the Sierra Madre del Sur, Trans-Mexican Volcanic Belt and the southern extreme of the Sierra Madre Oriental, giving rise to several endemic species, in addition to recolonizing South America.

Regarding the biome diversification, the DEC+J model fitted our dataset best (LnL = −134.35) (Fig. 2A), although there was no statistically significant difference compared with the DIVA+J model (LnL = −134.35). Both analyses yielded broadly similar results, with the most notable difference occurring at the crown node of sect. *Fecundae*: DEC+J inferred Tropical and Subtropical Moist Broadleaf Forest as the ancestral biome, whereas DIVA+J was ambiguous, suggesting either Tropical and Subtropical Moist Broadleaf Forest or Montane Grasslands and Shrublands. Throughout the phylogeny, Tropical and Subtropical Moist Broadleaf Forest emerged as the most probable biome for internal nodes, being prevalent across the cladogenesis of the three-stigmas clade. In contrast, the two-stigmas clade shifted entirely to Montane Grasslands and Shrublands from its crown node. Multiple independent colonizations of all biomes were detected. Biome specialization was particularly evident in the case of the well-supported clade comprising *Carex artoriasii* Lois *et al.*, *C. catrina* Lois *et al.*, *C. comolaflor* Lois *et al.*, *C. transmexicana* Lois *et al.* and *C. tuberculata* Liebm., whose diversification was linked to Temperate and Tropical Coniferous Forests. More than half of the species (20) are restricted to a single biome, and among these, eight are highly endemic, known only from their type localities.

### Bioclimate niche analysis

The Ornstein–Uhlenbeck (OU) model was selected as the best fit according to the corrected Akaike information criterion (AICc) [Brownian motion (BM): AICc = 558.48, log-likelihood = −277.04; OU: AICc = 557.07, log-likelihood = −275.14]. The evolution of the bioclimatic niche for the selected variables mapped onto the phylogeny is shown in Fig. 3. Overall, deeper nodes within the phylogeny of the section tended to show intermediate trait values, whereas extreme values were isolated towards the tips. Mean diurnal range, climatic moisture index and precipitation variables showed relatively homogeneous values within the two-stigma species that diverged more markedly from those observed at the three-stigma crown node and internal branches. The observed differences are merely observational. In the particular case of the aridity index, elevated values were located in endemic species of the Mexican mountain ranges (*C. tuberculata*, *C. transmexicana*, *C. comolaflor*, *C. catrina* and *C. artoriasii*), whereas most other species in the section exhibited intermediate values.

**Fig. 3.**
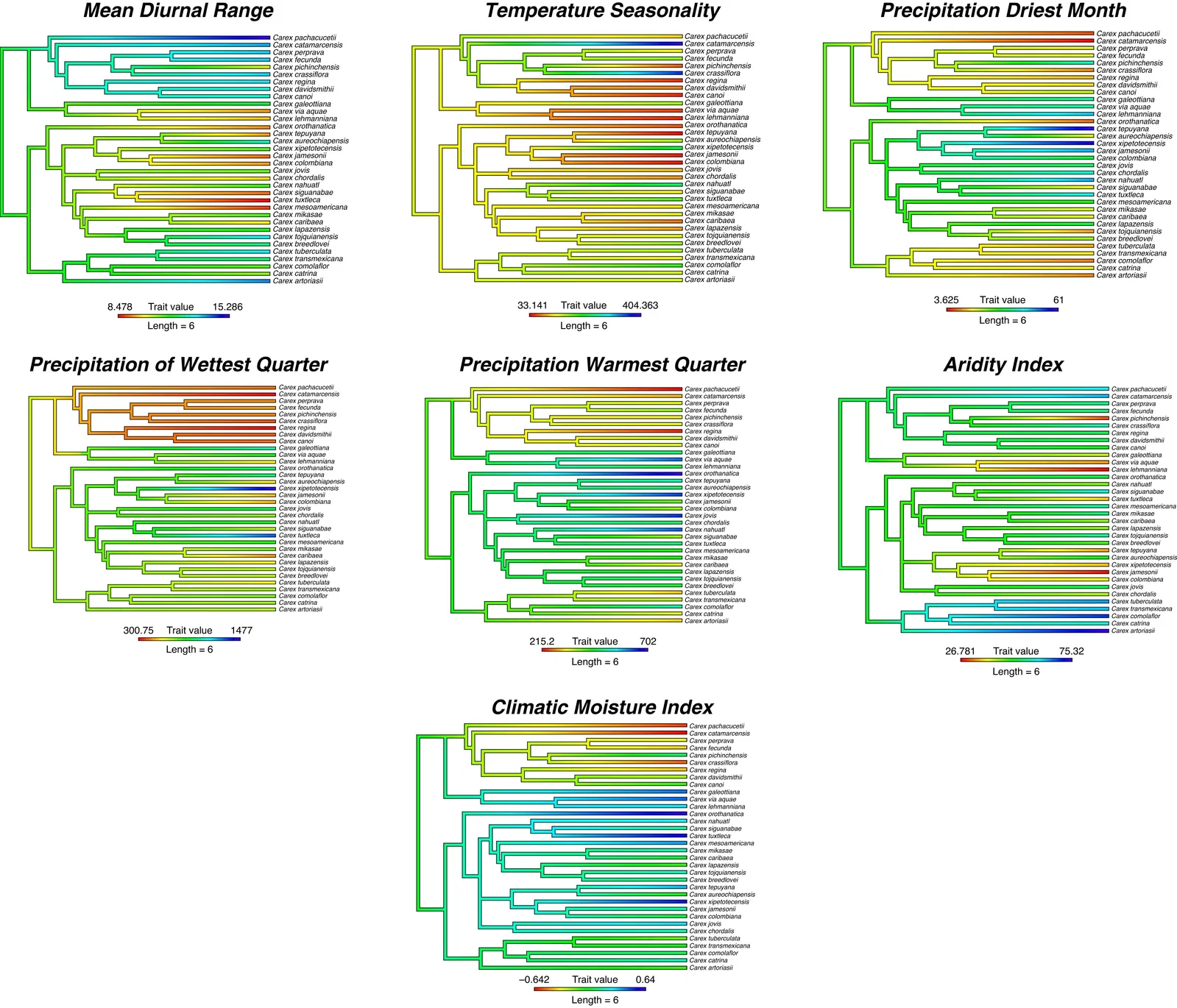
Bioclimatic niche reconstruction at ancestral nodes for four continuous climatic and environmental variables on the calibrated singleton tree for *Carex* sect. *Fecundae*. The reconstruction is represented as colour gradients along the branches. Higher values are displayed in blue, intermediate in green, and low in red.

### Ancestral state reconstruction of discrete characters

For nutlet invaginations, the best-fitting model was ARD, suggesting asymmetric transition rates between states. For the number of stigmas, ER was the best model, indicating symmetric rates between the two trait states.

A two-stigma utricle was inferred as the most probable ancestral state for *Carex* sect. *Fecundae* (Fig. 4A), with two well-supported transitions to three stigmas in *C. galeottiana* and at the base of the three-stigmas clade. With three stigmas as the apomorphic state in *Carex*, it implies a reversion to the ancestral condition from a two-stigmas ancestor.

**Fig. 4.**
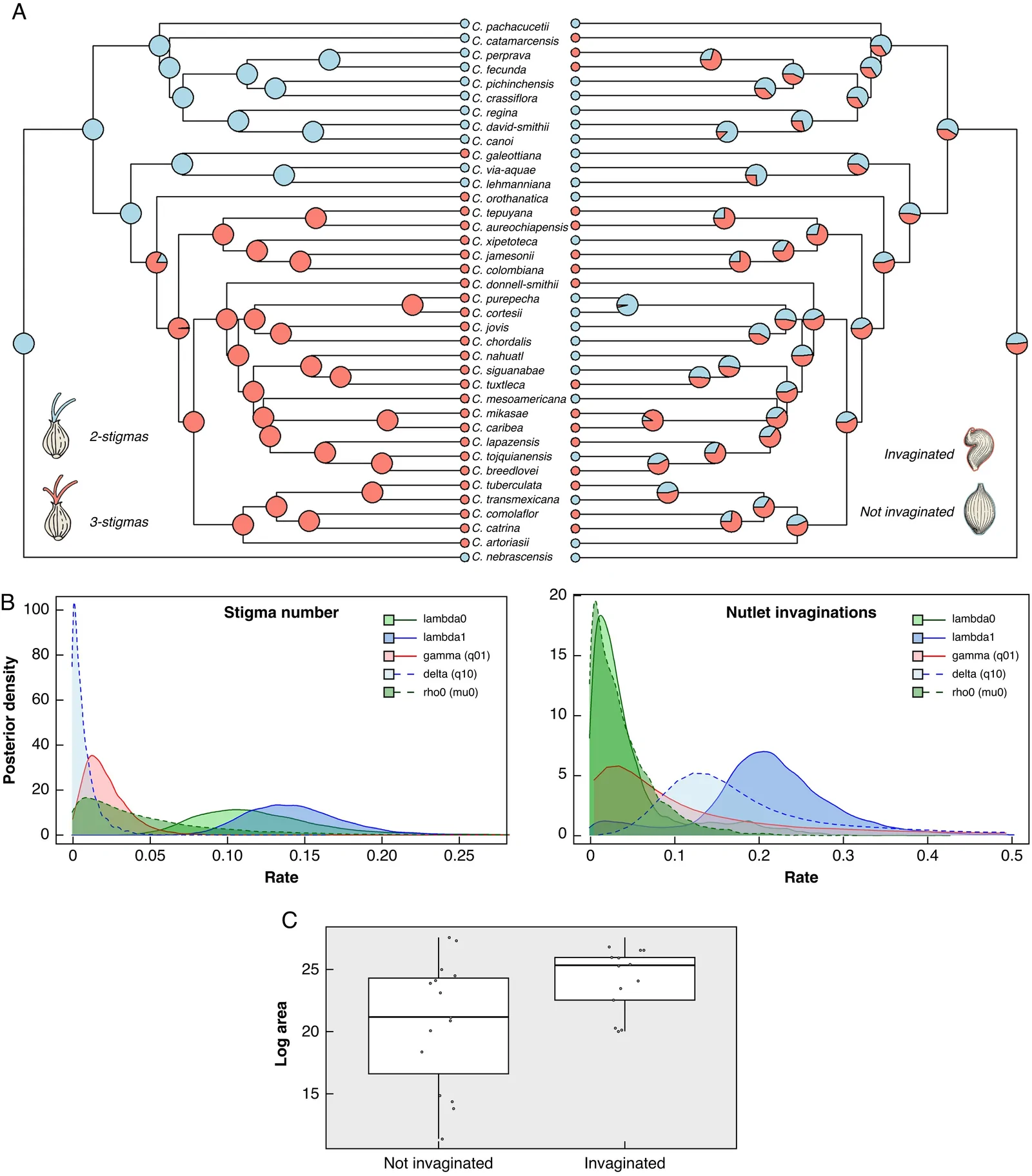
(A) Morphological state reconstruction for the number of stigmas (two or three) and shape of the nutlet (invaginated or not invaginated) for *Carex* sect. *Fecundae* using BiSSE. (B) Posterior probability densities of the estimated anagenetic parameters in the traits stigma number (left) and nutlet invaginations (right). The *x*-axis displays the rate of anagenetic parameters, and the *y*-axis indicates the posterior probability density of each value. (C) Boxplots comparing the area of occupancy between species with nutlet invaginations and species without invaginations. A PGLS test was used to assess significant differences between the two groups. Boxes represent interquartile ranges, and horizontal lines within the boxes indicate medians.

Regarding nutlet morphology, the presence of invaginations was inferred as the most likely ancestral state (Fig. 4A). Notably, transitions from invaginated to non-invaginated nutlets, in addition to reversions to the invaginated state, occurred multiple times independently across different supported clades.

### Evolution of continuous morphological traits

The OU model was selected as the best fit according to the AICc (BM: AICc = 418.6109, log-likelihood = −207.3054; OU: AICc = 415.8788, log-likelihood = −203.9394). The evolution of the selected variables mapped onto the phylogeny is shown in Fig. 5. As in the reconstruction of bioclimatic variables, deeper nodes within the phylogeny of the section tended to show intermediate trait values, whereas extreme values were isolated towards the tips. Only the number of spikes showed mostly homogeneous high values along all branches except on the terminals of *C. orotanathica* Lois *et al.* and *C. tojquianensis* Standl. & Steyerm., because most species of the section display many-flowered inflorescences except for these two species.

**Fig. 5.**
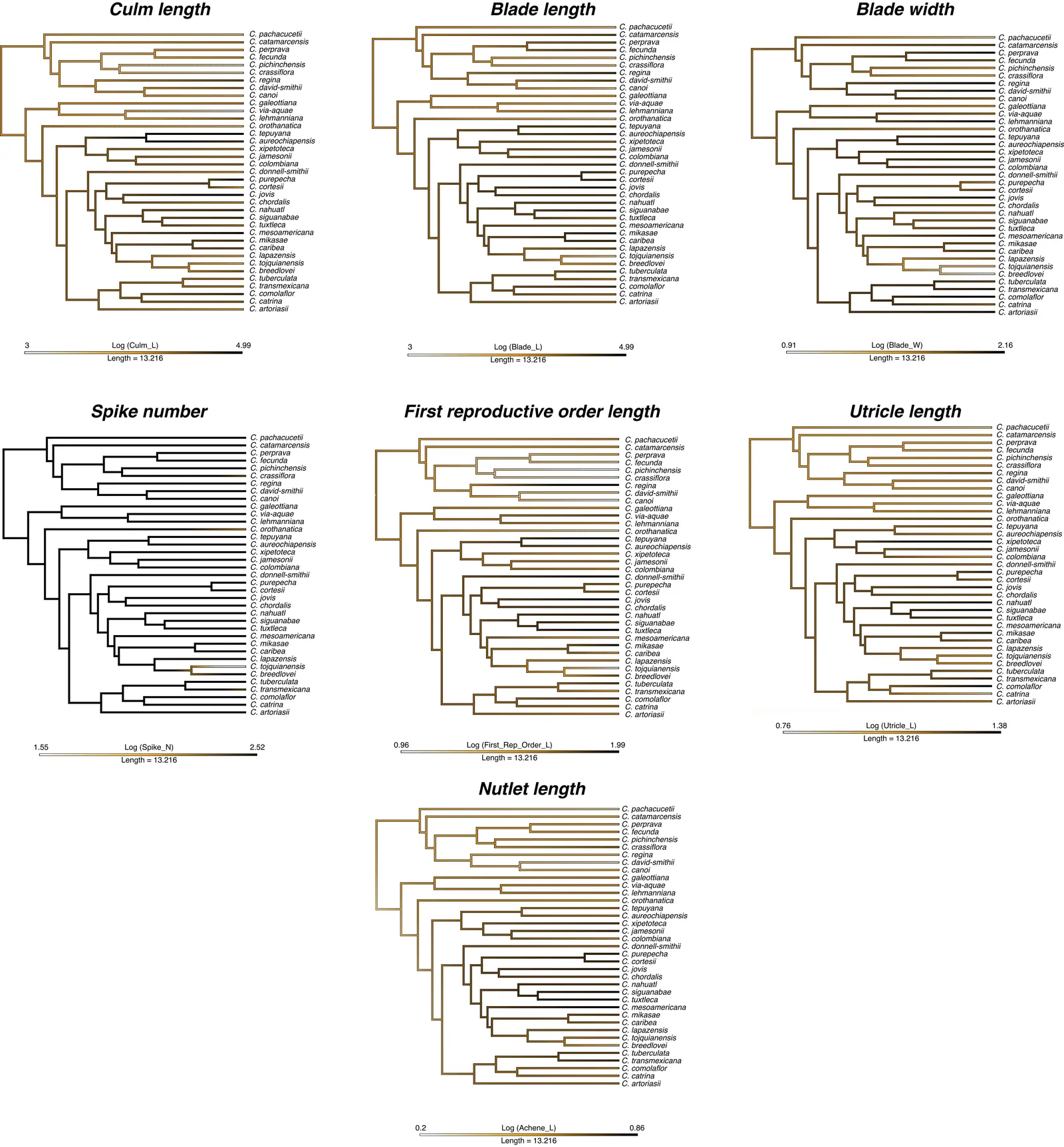
Reconstruction of the selected continuous variables on the calibrated singleton tree for *Carex* sect. *Fecundae*. The reconstruction is represented as colour gradients along the branches. Higher values are displayed in black, intermediate in brown, and low in white.

Out of all the pair comparisons, searching for correlation among continuous traits on the PGLS analysis, 12 of them presented a phylogenetic significant correlation (*P* < 0.05; Table 1; Supplementary Data S3). Of those, four explained extremely low variation (*R*^2^ < 0.1), two low variation (*R*^2^ = 0.1–0.3), three medium variation (*R*^2^ = 0.3–0.5) and three high variation (*R*^2^ = 0.5–0.6). The ones that showed the most variation were in the following pairs of traits: nutlet length versus utricle length; blade length versus blade width; and blade length versus culm length.

**Table 1.** Significant results (*P* < 0.05) from pairwise phylogenetic generalized least squares analyses between continuous traits. Each entry shows the coefficient of determination (*R*^2^) and the associated *P*-value, accounting for phylogenetic relationships.

Character 1	Character 2	*P*-value	*R* ^2^
Nutlet length	Blade width	0.0104	0.284
Nutlet length	Utricle length	0.000482	0.599
Blade length	Blade width	0.000113	0.555
Blade length	Culm length	0.000206	0.581
Blade length	First reproductive order length	0.00000456	0.321
Blade length	Spike length	0.000000566	0.001
Culm length	First reproductive order length	0.00772	0.273
Culm length	Spike number	0.00125	0.001
Culm length	Utricle length	0.00748	0.335
First reproductive order length	Spike number	0.000219	0.061
Flower number	Nutlet length	0.0396	0.064
Utricle length	Blade width	0.0251	0.318

Regarding the evaluation of phylogenetic signal among the continuous traits analysed, four (blade width, number of spikes, blade length and nutlet length) exhibited a strong phylogenetic signal (*P* < 0.05, *K* > 1 and λ ≈ 1). Utricle length showed a discrepancy between metrics, with a significant *K* value (*K* = 1.16, *P* = 0.004) but a moderate λ (λ = 0.59), suggesting partial phylogenetic structure. The remaining traits (first-order spike length, culm length and number of flowers) did not exhibit a phylogenetic signal (*P* = 1, *K* ≪ 1 and λ ≈ 0).

### Diversification analyses

The BAMM analysis achieved convergence after discarding the first 10 % of samples as burn-in. No shifts in net diversification rates were detected; instead, the highest diversification rates were achieved at the origin of the section, and diversification rates decelerated towards the present, when the lowest diversification rates were displayed.

Results from both BiSSE and HiSSE analyses indicated that the best-fitting model was the two-parameter BiSSE model without hidden states (Table 2). This indicates that diversification rates are character dependent and associated with the traits analysed, namely, number of stigmas and the presence of invaginations in the nutlet. The absence of support for hidden states suggests that the observed traits alone sufficiently explain the diversification dynamics.

**Table 2.** Model comparison results for BiSSE and various HiSSE models assessing the association between diversification rates and two binary traits: number of stigmas and presence of achene invaginations. Shown are the log-likelihood (logL), number of parameters (*k*), and Akaike information criterion (AIC) for each model.

Model	logL	*k*	AIC
**Number of stigmas**			
4-state hidden rate model	−26.21613	6	64.4323
Two stigma hidden	−26.40972	4	60.8194
Three stigma hidden	−26.25421	4	60.5084
No hidden states (BiSSE)	−26.40972	2	56.8194
**Nutlet invaginations**			
4-state hidden rate model	−26.2197	6	64.4394
Not invaginated	−26.25421	4	60.5084
Invaginated	−26.40972	4	60.8199
No hidden states (BiSSE)	−26.40972	2	56.8194

Fig. 4B shows the posterior density distributions of key parameters used to analyse the evolution of two discrete traits: nutlet invagination and number of stigmas. The parameters are: ρ_0_, the extinction rate for state 0; γ and δ, the transition rates between trait states (q01 and q10, respectively); and λ_0_ and λ_1_, the speciation rates for states 0 and 1, respectively.

For nutlet invagination, the posterior distribution peaked at ρ_0_, indicating that differences in diversification are better explained by variation in extinction rates between invaginated and non-invaginated states, reflecting distinct phylogenetic patterns. The estimated speciation rates show that λ_1_ (invaginated nutlets) is higher than λ_0_ (not invaginated nutlets), indicating faster diversification in species with invaginated nutlets.

In contrast, the posterior distribution for stigma number peaked at δ, indicating that diversification between species with two versus three stigmas is driven primarily by asymmetric transition rates between these states, consistent with trait evolution towards two distinct adaptive optima. Despite the smaller differences in λ values for the number of stigmas in comparison to those observed for nutlet invaginations, both traits show distinct evolutionary dynamics, with different estimated values for λ_0_ and λ_1_. Correspondingly, λ_1_ (three-stigmas) is higher than λ_0_ (two-stigmas), indicating a higher speciation rate in species with three stigmas.

These results reveal contrasting evolutionary dynamics between the two traits, with nutlet invagination diversification influenced mainly by extinction differences linked to phylogenetic patterns, and stigma number diversification driven primarily by trait transition dynamics and adaptive divergence.

### Evaluation of nutlet invagination and area of occupancy

We performed phylogenetic logistic regression analyses to assess the relationship between nutlet invagination (binary trait) and species distribution area (log-transformed and standardized). Using the logistic_IG10 method, the model estimated a positive association between distribution area and the presence of nutlet invagination (estimate = 0.97, standard error = 0.53), indicating a marginally significant trend (*P* = 0.064).

Likewise, the logistic_MPLE method produced a comparable positive effect size (estimate = 0.91, standard error = 0.52), with a marginal *P*-value (*P* = 0.079).

Both models indicated moderate phylogenetic signal (α parameters of 0.088 and 0.131, respectively), suggesting that evolutionary history influences the trait distribution. Fig. 4C presents boxplots for illustrative purposes, providing a clearer visualization of the data distribution.

## DISCUSSION

### First phylogenomic insights into *Carex* sect. *Fecundae*

Prior to this study, no exhaustive phylogeny of *Carex* sect. *Fecundae* had been published. Earlier phylogenetic analyses included only a few representatives of the section (e.g. Hendrichs *et al.*, 2004; Martín-Bravo *et al.*, 2019), providing limited insights. Our results deliver the first comprehensive phylogenomic reconstruction of this previously neglected Neotropical group, sampling 95 % of known taxa. Our findings confirm the monophyly of the section if *C. hebetata* is excluded. *Carex* sect. *Fecundae* is nested within an evolutionary grade formed by North American *Carex* species usually placed within sect. *Phacocystis* Dumort. This pattern suggests that sect. *Phacocystis*, as broadly circumscribed, might represent a paraphyletic assemblage rather than a strictly monophyletic group. In this context, sect. *Fecundae* appears as a well-defined lineage embedded within that broader grade.

The internal phylogenetic pattern recovered an overall correspondence with stigma number. The species with two stigmas form a grade towards clade 3, composed only of three-stigma taxa (Fig. 1). Beyond these points of agreement, few comparisons with prior research are possible owing to the historical neglect of sect. *Fecundae*. Future studies including broader sampling of species traditionally assigned to sect. *Phacocystis* will be necessary to clarify the limits of sect. *Phacocystis s.s.* and to evaluate the most appropriate taxonomic treatment of these lineages.

We resolved the monophyly of several widely distributed and/or morphologically complexes and species. On the one hand, the included populations of *C. donnell-smithii* L.H. Bailey and *C. chordalis* Liebm., species that extend from Mexico to South America, consistently recovered each species as monophyletic. On the other hand, species of the ‘tuberculata complex’, comprising *C. catrina*, *C. comolaflor*, *C. tuberculata*, *C. transmexicana*, *C. cortesii* Liebm. and *C. artoriasii* (Lois *et al.*, 2025), are each strongly supported (>98 % BS). *Carex fecunda* Steud., a vaguely characterized taxon, was also recovered as monophyletic but not with the highest support (>90 % BS) from the closely related *Carex perprava* C.B. Clarke. This, together with the deep branches found within the included populations of *C. fecunda*, point to the need for additional study within this clade.

Despite the strong bootstrap support across the tree, some internal nodes, especially along the backbone, exhibited low gene and site concordance factors, notably gCF. This pattern suggests a complex history that might include incomplete lineage sorting and/or past hybridizations, frequently tied to the dynamic Neotropical environment (Maddison and Lacey, 2006; Joly *et al.*, 2009). Putative recent polyploidy has recently been suggested for subgenus *Uncinia* (García-Moro *et al.,* in press) and might also be playing a role in the internal disruption of the involved clades. Nevertheless, polyploidy is a rather infrequent biological process in genus *Carex*, affecting only some clades (Shafir *et al.*, 2023), with high rates of fusion and fission being the major genomic driver of diversification in genus *Carex* (Escudero *et al.*, 2012; Tribble *et al.*, 2025).

In addition, diversification analyses based on BAMM indicate a gradual decline in diversification rates through time, which does not support the occurrence of rapid adaptive radiations. Instead, these results are more consistent with a pattern of species accumulation through time, potentially associated with *in situ* speciation in certain regions.

The exclusion of *C. hebetata* from sect. *Fecundae* is somewhat surprising yet not entirely unexpected. This Peruvian endemic shows dark-coloured many-spiked inflorercences, like most members of sect. *Fecundae*. However, this is not an exclusive character of the section, but a combination of characters somewhat recurrent in *Carex* species that transition into Tropical ecosystems (see Roalson *et al.*, 2021). Surprisingly, *C. hebetata* has a key character highly conserved within sectional lineages that is not shared with sect. *Fecundae*: the lowermost bract is sheathing, whereas in sect. *Fecundae* it is not. This was noticed already by Kükenthal (1909), but he was misled by the striking resemblance of the floral parts.

### Biogeographical history of *Carex* sect. *Fecundae*

Section *Fecundae* is unique within *Carex* in that it is the only clade entirely restricted to tropical Americas, where it is confined to montane and high-elevation habitats (Roalson *et al.*, 2021; Lois *et al.*, 2025). Its biogeographical history is closely tied to the elevational uplift of Neotropical mountain ranges, during which new ecological opportunities for colonization were generated.

Our biogeographical reconstructions (Fig. 2A) suggest that *Carex* sect. *Fecundae* originated in the Central Andes during the Late Miocene from a North American ancestor via a long-distance dispersal event, probably facilitated by the advanced development of the Andes at ∼10 Mya (Gregory-Wodzicki, 2000). In general, the early diversification of the group occurred during the global cooling of the Miocene (Herbert *et al.*, 2016), which helped cold-adapted lineages to expand and diversify worldwide (Estep *et al.*, 2014; Steinthorsdottir *et al.*, 2021). This suggests that sect. *Fecundae* might have been somewhat pre-adapted to the new habitats created by the combined action of global cooling and mountain uplift. Remarkably, diversification rates in sect. *Fecundae* appear to decrease progressively over time (Fig. 2C), a pattern that aligns with other Neotropical sections of *Carex* (Benítez-Benítez *et al.*, 2021*a*, *b*), but contrasts with the diversification dynamics observed in the genus as a whole (Martín-Bravo *et al.*, 2019). This characterizes the observed diversity of the group as a disparification (Simões *et al.*, 2016) rather than as a radiation. It means that the current species richness within the section would be attributable primarily to the gradual accumulation of evolutionary changes over time towards the present and probably little extinction. Most cladogenetic events coincide temporally with major phases of Andean uplift throughout the Miocene and Pliocene (Gregory-Wodzicki, 2000; Hartley, 2003; Antonelli *et al.*, 2009). Our results indicate limited cladogenesis during the Pleistocene, a period marked by strong climatic instability (Pisias and Moor, 1981). This pattern aligns with the notion that tropical lineages, such as *Carex* in the Neotropics, are more dependent on stable environmental conditions and less prone to diversification under fluctuating climates.

Our results support a scenario in which sect. *Fecundae* reached the Southern Andes via long-distance dispersal from North America, and clade 1 diversified entirely within high-altitude grasslands and shrublands of South America (Fig. 2B). During this process, the group seemingly underwent changes in the bioclimatic preferences related to precipitation variables (Fig. 3), probably reflecting adaptation to the much drier climates found on the western slopes of the Andes (Insel *et al.*, 2009). This contrasts with other species of the section inhabiting areas with more intermediate precipitation. Local adaptation seems to be suggested by the heterogeneity observed for temperature seasonality values, probably as result of isolation within allopatric glacial refugia (Sales and Pires, 2023).

The origin and diversification of clades 2 and 3 support a scenario in which sect. *Fecundae* moved progressively northwards from the Central Andes (Fig. 2A). This might have been facilitated by the late stages of uplifting of the Cordillera Centroamericana and Sierra de Talamanca (Kobayashi *et al.*, 2014), which happened between 10 and 5 Mya (Morell *et al.*, 2012). The early shifts into tropical montane environments (such as montane grasslands, shrublands and moist forests) seen in the group (Fig. 2B) further support the idea that sect. *Fecundae* lineages occupied these new niches as they became available. Additional evidence of pre-adaptation is provided by the remarkable long-term niche conservatism in variables such as mean diurnal range and precipitation variables (Fig. 3). Colonization of cold-adapted groups related to orogenetic processes in Tropical America has been reported in other plant groups in the Andes (Antonelli *et al.*, 2009), Central America (Kobayashi *et al.*, 2014) and Mexico (Pardo and Suárez, 1995; Bryson *et al.*, 2011).

The colonization of the Mexican highlands (the northernmost limit of the section) happened more recently (Fig. 2A), in connection with the uplift of the Trans-Mexican Volcanic Belt (Marshall and Liebherr, 2000; Rosas-Elguera *et al.*, 2003), the highest mountain range in Mexico. Historically, these areas were lower (<3000 m) and had warmer climates. Their current altitudes were reached during the Pliocene, which coincides with the diversification of the Mexican endemic sect. *Fecundae* species. It is during this time when the group underwent its latest ecological transition and colonized the Temperate and Tropical Coniferous Forests (Fig. 2B) that occupied this area synchronically (Sosa *et al.*, 2018). In Mexico, sect. *Fecundae* diversified into several narrowly endemic species, probably driven by the isolation of suitable high-elevation habitats as sky islands (Morawetz and Raedig, 2007; Philbrick *et al.*, 2010). One striking case is *Carex tuxtleca* Lois *et al.*, endemic to the small, coastal volcanic Sierra de los Tuxtlas, which is an isolated mountain completely surrounded by tropical lowlands and unconnected to any other Mexican ranges. Shifts in bioclimatic preferences do not seem to have played a major role in the Mexican diversification of the sect. *Fecundae*, because intermediate bioclimatic and environmental values prevail before the shallowest phylogenetic levels of the involved clades (Fig. 3).

Importantly, although some climatic niche variables show overall conservatism, our results reveal that climatic niche patterns within sect. *Fecundae* are not entirely homogeneous. For example, preferences for drier environments, as seen in the Mexican clade (*C. artoriassi*, *C. catrina*, *C. comolaflor*, *C. transmexicana* and *C. tuberculata*), might point to local adaptation in geographical restricted species. This appears to have influenced recent diversification and highlights aridity as a key ecological factor driving diversification within the section (Fig. 3).

### The role of two enigmatic morphological characters in the evolution of *Carex* sect. *Fecundae*

Two of the most remarkable features found in sect. *Fecundae*, the number of stigmas and nutlet invaginations, do not display clear functional relevance in the evolution of the group. However, our analyses show that they have an impact on its diversification (Table 2). The first feature is confined to particular sets of lineages, whereas the second is an evolutionarily dynamic feature that seemingly transitioned several times from one state to another (Fig. 4A).

The prevalence of two stigmas across the South American clade 1 and the change to three stigmas in clade 3 (Fig. 4A) support evolutionary transitions to different diversification optima. Remarkably, the climatic niche associated with two-stigma species corresponds to drier environmental conditions, whereas three-stigma species tend to occupy regions with moderate to high precipitation levels (Fig. 3). In the context of wind pollination, a higher number of stigmas might ease the binding of pollen (Chaves *et al.*, 2024). Given the high number of flowers of most sect. *Fecundae* species (Lois *et al.*, 2025), the addition of one additional stigma could result in a notable increase in the amount of pollen-catching surface in each inflorescence. In the wetter climates, where species of clade 3 dwell, this additional stigma might play a role facilitating pollination. On the contrary, the drier climates that species of clade 1 inhabit might favour the maintenance of two stigmas. Given that the stigma is the most delicate and least protected female organ exposed to adverse or extreme environmental conditions (Fábián *et al.*, 2019), maintaining a lower number of stigmas in xeric conditions also entails risking less resources in flowering.

However, the evolutionary drivers underlying variation in the number of stigmas remain largely cryptic. Two-stigma conditions occur in several other lineages of *Carex*, particularly within sect. *Phacocystis*, often in taxa adapted to cold or stressful environments, such as lakeshores, swamps, bogs or high-altitude and (sub-)Arctic habitats. Therefore, although the occurrence of two stigmas in clade 1 might reflect a putative selective advantage in certain environmental conditions, the underlying evolutionary mechanisms remain uncertain.

Regarding nutlet morphology, ancestral state reconstruction supported the presence of invaginations as the most likely ancestral condition within the sect. *Fecundae*. Although no clear relationship was found between bioclimatic niche and nutlet invaginations; our phylogenetic logistic regression analyses revealed a marginally significant positive association between the presence of nutlet invaginations and species area of occupancy. This recurrent pattern of trait loss and subsequent recovery suggests functional significance, indicating convergent evolutionary responses within the section. This strongly suggests a potential ecological function for nutlet morphology in dispersal or establishment. Although the taxonomic value of the invagination in nutlets has long been known in *Carex* (e.g. Kükenthal, 1909; Jiménez-Mejías and Martinetto, 2013), little has been discussed about the role of this feature. Ant-mediated seed dispersal (myrmecochory) has been widely reported in *Carex* (Handel, 1976; Kjellsson, 1985; Tanaka and Tokuda, 2020); in particular, several species from sections *Clandestinae* and *Mitratae* possess a thickened, protein-rich basal portion of the utricle resembling an elaisome that attracts ants, although such structures have not been reported in species of section *Fecundae*.

In this context, the invaginated nutlet shape observed in sect. *Fecundae* might represent a different or complementary feature potentially influencing interactions with ants or other insects. Perhaps the invaginations might facilitate attachment or manipulation by ants or other insects, enhancing dispersal efficiency, promoting the successful colonization of suitable microhabitats and reducing negative density-dependent interactions between seeds and conspecific adults (Tanaka and Tokuda, 2020). Speculatively, another possible interpretation of the curved shape of the nutlet would be mechanical protection of the embryo or facilitating a particular anchoring to the substrate. Many species grow in ravines and on moist, steep slopes, where good anchoring is particularly advantageous against surface runoff. Although not tested, it is conceivable that any of these hypotheses could contribute to increasing seedling survival and establishment and, in turn, to a broader geographical distribution. The two most widespread species of the section, *Carex chordalis* and *C. donnell-smithii*, which range from Central Mexico to South America, often have invaginated nutlets; in the second, nutlets can even be completely irregular. In addition, we cannot rule out a role of the invagination in processes of epi- or endozoochory, which are known in other *Carex* groups (Villaverde *et al.*, 2017) and that could effectively facilitate the broader observed distributions. In any case, experimental fieldwork would be desirable to test the role of nutlet invagination in the reproductive history of *Carex*.

### Building titans: evolution of size in the giant sect. *Fecundae*

As expected, the traits that exhibited the strongest correlations were those whose sizes are typically interdependent within the genus *Carex* (Table 1), notably the positive correlations between the length of the utricle and the nutlet, blade length and width, and blade length and culm length. An additional noteworthy finding is the positive correlation between utricle and nutlet length with blade width. This suggests that the evolution of size in sect. *Fecundae* has a similar impact on different organs. Remarkably, the South American clade tends to display consistently lower values in culm, blade, nutlet and utricle length (Fig. 5), a group that was also related to low values on precipitation variables (Fig. 3). This might point to size being affected by the availability of resources, as reported in other sedge groups (Léveillé-Bourret *et al.*, 2020). The lack of correlation of blade length with the other traits is probably attributable to the bias in collection of the measured specimens of sect. *Fecundae*; rarely, the longest leaf is going to be processed with the voucher, but rather probably discarded.

Remarkably, we observed contrasting levels of phylogenetic signal among the morphological traits analysed. Although most traits directly related to size displayed a strong phylogenetic signal, suggesting phylogenetic conservatism, utricle length displayed an intermediate phylogenetic signal (Pagel’s λ = 0.595103), suggesting that although closely related species tend to exhibit somewhat similar utricle lengths, substantial variation exists. This is especially striking when one considers that nutlet length exhibited a high phylogenetic signal (Pagel’s λ ≈ 1), indicating that utricle and nutlet size might evolve separately. This could reflect selective pressures acting on the utricle but not on the nutlet it contains, because the utricle is the organ that has the important task of dispersal in *Carex* (Jiménez-Mejías *et al.*, 2016*b*; Martín-Bravo *et al.*, 2019).

### Conclusion

Our study reveals that *Carex* sect. *Fecundae* represents a remarkable case of tropical diversification within a predominantly boreotemperate genus. The section displays a pattern of diversification linked to the orographic history of the Neotropical mountains, with phylogenomic evidence supporting its monophyly and uncovering previously undetected relationships among species. The colonization of different biomes underscores the role of ecological transitions in shaping its current diversity. Trait evolution analyses suggest divergent evolutionary dynamics among reproductive structures (such as the number of stigmas, nutlet morphology, and utricle and nutlet size), highlighting the importance of these organs in the evolutionary history of the group. Unexpectedly, nutlet invaginations were associated with larger distribution ranges, although their biological relevance remains unclear and warrants further investigation.

Altogether, the evolutionary trajectory of sect. *Fecundae* illustrates how geological uplift, climatic shifts, morphological innovation and long-distance dispersal have acted synergistically to drive diversification and ecological adaptation in a cold-adapted lineage within tropical montane environments. This work sheds light on the processes underlying the exceptional biodiversity of tropical mountain ecosystems and provides a solid phylogenetic and ecological framework for future taxonomic, evolutionary and conservation studies in *Carex*.

## Supplementary Material

mcag177_Supplementary_Data

## SUPPLEMENTARY DATA

Supplementary data are available at *Annals of Botany* online and consist of the following. Table S1: comparison of evolutionary models for each morphological trait analysed, including Brownian motion (BM), Ornstein–Uhlenbeck (OU) and λ models. The Akaike information criterion (AIC) values are presented for each model per trait. Data S1: studied material included in the phylogenomic study. Data S2: BioGeoBEARS results for the tested models, showing reconstructions of both biogeographical areas: (A1) four-area DEC; (A2) four-area DEC+J; (A3) four-area DIVA; (A4) four-area DIVA+J; (A5) eight-area DEC; (A6) eight-area DIVA; (A7) eight-area DIVA+J; and biomes: (B1) DEC; (B2) DIVA; and (B3) DIVA+J. Data S3: Scatterplots showing significant linear relationships between continuous traits based on phylogenetic generalized least squares (PGLS) analyses. Each plot displays the regression line, the coefficient of determination (*R*^2^) and the *P*-value, accounting for phylogenetic non-independence.
